# Surgical reporting for laparoscopic cholecystectomy based on phase annotation by a convolutional neural network (CNN) and the phenomenon of phase flickering: a proof of concept

**DOI:** 10.1007/s11548-022-02680-6

**Published:** 2022-05-28

**Authors:** M. Berlet, T. Vogel, D. Ostler, T. Czempiel, M. Kähler, S. Brunner, H. Feussner, D. Wilhelm, M. Kranzfelder

**Affiliations:** 1 Department of Surgery, Klinikum rechts der Isar, Technical University of Munich, Munich, Germany; 2 Research Group Minimally-Invasive Therapy and Intervention (MITI), Klinikum rechts der Isar, Technical University of Munich, Munich, Germany; 3 Chair for Computer Aided Medical Procedures and Augmented Reality (CAMP), Technical University of Munich, Munich, Germany

**Keywords:** Computer aided operative notes, Surgical documentation, Machine learning, Laparoscopic cholecystectomy

## Abstract

**Purpose:**

Surgical documentation is an important yet time-consuming necessity in clinical routine. Beside its core function to transmit information about a surgery to other medical professionals, the surgical report has gained even more significance in terms of information extraction for scientific, administrative and judicial application. A possible basis for computer aided reporting is phase detection by convolutional neural networks (CNN). In this article we propose a workflow to generate operative notes based on the output of the TeCNO CNN.

**Methods:**

Video recordings of 15 cholecystectomies were used for inference. The annotation of TeCNO was compared to that of an expert surgeon (HE) and the algorithm based annotation of a scientist (HA). The CNN output then was used to identify aberrance from standard course as basis for the final report. Moreover, we assessed the phenomenon of ‘phase flickering’ as clusters of incorrectly labeled frames and evaluated its usability.

**Results:**

The accordance of the HE and CNN was 79.7% and that of HA and CNN 87.0%. ‘Phase flickering’ indicated an aberrant course with AUCs of 0.91 and 0.89 in ROC analysis regarding number and extend of concerned frames. Finally, we created operative notes based on a standard text, deviation alerts, and manual completion by the surgeon.

**Conclusion:**

Computer-aided documentation is a noteworthy use case for phase recognition in standardized surgery. The analysis of phase flickering in a CNN’s annotation has the potential of retrieving more information about the course of a particular procedure to complement an automated report.

## Introduction

As surgeons worldwide are legally obligated to provide prompt, detailed, thorough, and complete documentation of surgical procedures (surgical reporting) [1, 2], each approach facilitating an easy to create, yet comprehensive documentation is noteworthy.

Literature search revealed several studies showing that unstructured and handwritten operative notes are often poor in quality, but interventions like training with the intent to enhance structure, did result in significant improvement. [3, 4] By now, most surgeons make use of templates for documentation as this can verifiably improve quality. [5] In addition, structured operative notes with a predefined wording empower automated information extraction (IE) for economic and scientific applications apart from a bare medical significance. [6] An obvious approach to foster a reliable yet applicatory surgical reporting is the use of computer science.

Recent developments paved the way to stunning new applications. The wide field of Machine Learning (ML), especially the use of convolutional neural networks (CNN) has taken video, audio, and speech recognition a giant step further. [7] For instance, machine learning enables the extraction of adverse events from narrative surgical reports today [8]. The logical consequence is to construct applications that allow to create even comprehensive surgical documentation based on this technique. The most significant modality regarding ML in surgery is the image and video recognition as it facilitates the detection of phases and instruments. [9] EndoNet was one of the first CNNs enabling multiple recognition tasks in videos of laparoscopic cholecystectomies [10]. Its annotations were based on 8 phases (P0−P7) specified in the Cholec80 data set. The authors mentioned a possible application in real-time surgical scheduling and automatic indexing of video databases. However, the information gained from highly reliable phase annotation by CNNs can not only be used for real-time applications but also for the much less time-critical generation of operative notes. In 2020, Czempiel et al. introduced a CNN called TeCNO which incorporates the timely course of a laparoscopic cholecystectomy producing a smoother phase recognition. [11] As our research group was significantly involved in the development and training of this project contributing medical expertise and recordings of surgeries, we now apply the resulting CNN to a specific medical issue. Phase annotation thereby seems to be a proper approach to the challenge of structuring a surgical procedure. [12] Admittedly, this technique seems artificial or even arbitrary in some cases as different surgeons may adhere to a divergent order of surgical steps. Nevertheless, many critical events as ‘clipping an artery’ or ‘dissection of the gallbladder’ are milestones that must be completed to reach the intended surgical result, and allow a robust phase definition. Even classic handwritten surgical notes are mainly structured on the basis of these critical steps according to our experience in daily clinical practice. In this original research article we propose a first proof of concept allowing partly automated surgical reporting for laparoscopic cholecystectomy by a CNN output. We use phase annotations of 15 cholecystectomies delivered by TeCNO after a training with 52 surgeries recorded in our surgical department, and try to retrieve information from these datasets that could serve as a basis for a reliable and easy to apply reporting tool. Moreover, we explore, how patterns of incorrectly assigned frames (‘phase flickering’) in the CNN’s annotation can be used to obtain even more information, e.g. about adverse events for an automated report. To evaluate validity of annotations and the subsequent operative notes, we compare the TeCNO results with that of an experienced surgeon (HE) and an algorithm-based annotation by a human scientist (HA). Finally, we propose a workflow to generate feasible operative notes based on the data obtained by the use of a self-programmed reporting tool.

## Methods

### Study design

The videos used for the study were recorded during routine laparoscopic cholecystectomies in our Department of Surgery at the Klinikum rechts der Isar, Technical University of Munich. All patients gave their written informed consent according to the vote of the local ethics committee. Firstly, the CNN was trained with 52 videos of routinely recorded cholecystectomies. Then 15 further videos were used for inference with a resolution of 2 frames per second. Eight phases have been defined previously based on the Cholec80 dataset: Phase **P0**: Transition start, Phase **P1**: Preparation, Phase **P2**: Clipping, Phase **P3**: Dissection, Phase **P4**: Hemostasis part I, Phase **P5**: Retrieval of the gallbladder, **P6**: Hemostasis part II, **P7**: Transition end. Independently of the phase detection by the convolutional neural network (CNN), a surgeon with an experience of more than 10,000 cholecystectomies (Human Expert, HE) annotated the 15 videos based on the same 8 phases. In addition, a non-experienced human scientist (Human Algorithm based, HA) also annotated the videos according to a predefined algorithm. Finally, the results of the CNN and HA annotation were compared with the experienced surgeon’s annotation committed as ground truth. The neural network annotation results were then used to create a detailed surgical report in interaction with the theoretical surgeon based on standardized text snippets. The reports were generated using the MITI Surgical Report tool version 1.1, which was written by the authors themselves from the scratch exclusively for this purpose. The duration of a given phase served as indicator to detect the accordance to the assumed standard course. The surgeon receives a warning message in the Graphic User Interface (GUI), if a deviation from the standard length of one or more of the 8 phases was detected. Otherwise, the default text snippet for that particular phase is used for the final output. The MITI Surgical Report tool was written in the PHP language and is available as an online application.

**Fig. 1:**
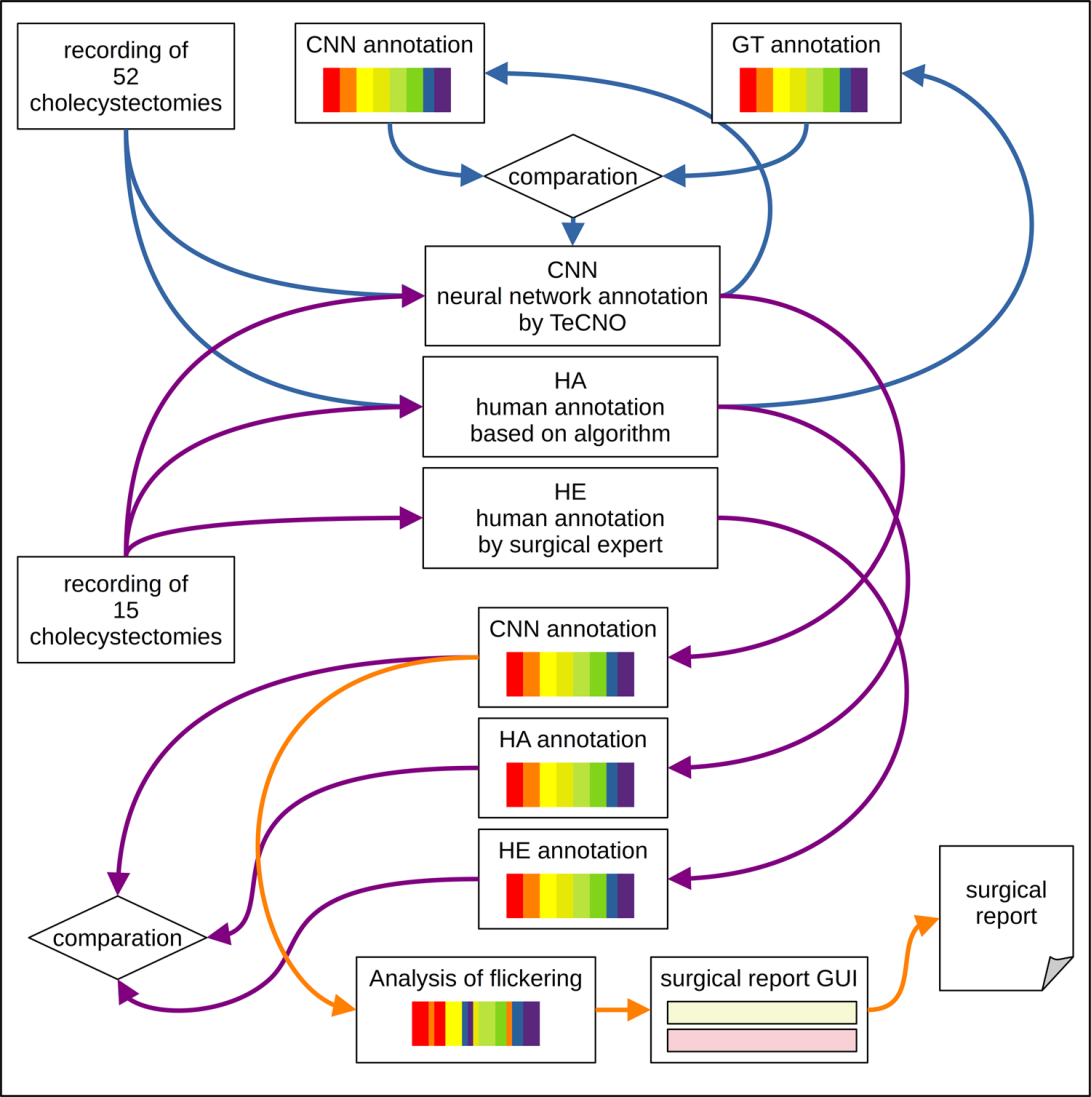
Workflow of the study – Blue arrows: training process (*n* = 52), Purple arrows: annotation process of the study collective (*n* = 15), Orange arrows: Analysis of phase flickering and creation of the surgical report with the self written MITI Surgical Report tool. CNN: neural network TeCNO, HA: Human annotator algorithm based, HE: Human annotator surgical expert, GT: ground truth

To gain further depth of information we additionally analyzed the patterns of incorrect respectively illogically assigned frames during phase recognition as we noticed, that especially in surgeries exceeding the IQR of phase durations this phenomenon of ‘phase flickering’ was dominant. The main idea was to identify surgeries with adverse events like bleeding or technical problems more precisely. The entire workflow of the study is depicted in Fig. 1.

### Statistical evaluation

Statistical analysis of the results was performed using the statistical framework R, version 3.6.3 [13] with addition of the packages *caret* version 6.0–90 [14], *ggplot2* version 3.3.5 [15], *e1071* version 1.7–9 [16], *lattice* version 0.20–45 [17], and *pROC* [18]. Endpoints were accuracy (ACC) regarding the annotation accordance of the CNN and the HA compared with that of the HE and the accordance in alert behavior during the generation of the final reports. To visualize the deviation of the CNN and HA results from those of the HE, confusion maps with the ground truth in columns and prediction in rows were used for each phase. To evaluate the accuracy of the CNN and the HA compared with the gold standard HE, all 15 annotations were merged and the correct assignment of each frame to one of the 8 given phases was considered in the statistics.

Approaching the phenomenon of ‘phase flickering’ during annotation by the CNN, we extracted frame clusters of disordered assignment with frequent changes of annotated labels without a clear phase transition and analyzed the affected videos for possible adverse events related to impeded recognition. Furthermore, we assessed the number of those groups with flickering or transitions (FT groups) and the amount of concerned frames with a Receiver Operator Characteristic (ROC) to define possible cutoff values for the decision for or against a possible aberrant course and consequently an alert in the final reporting software.

### Creation of the final output

The phase durations of all 15 cholecystectomies used in this study served as the basis for the decision for or against an aberrant course. If a phase duration deviated from the Interquartile Range (IQR) of the entire collective, a warning message was issued to the GUI in form of a red colored background behind the box containing the standard text for the respective phase. This alert advised the surgeon to change the standard text in order to comment on the deviation, e.g. in case of extended preparation time due to intraoperative bleeding. The alarm was triggered when the duration of the respective phase was lower than the 25th quantile or exceeded the 75th quantile. Thus, two outcomes per phase were possible. Either an alert was triggered in case of duration suspect for an adverse event and deviation from standard course or the text field was colored green in case of a phase duration within the IQR. The warning behavior of the system was then evaluated both based on the annotation of the CNN and of the human expert (HE).

## Results

### Phase detection

**Fig. 2:**
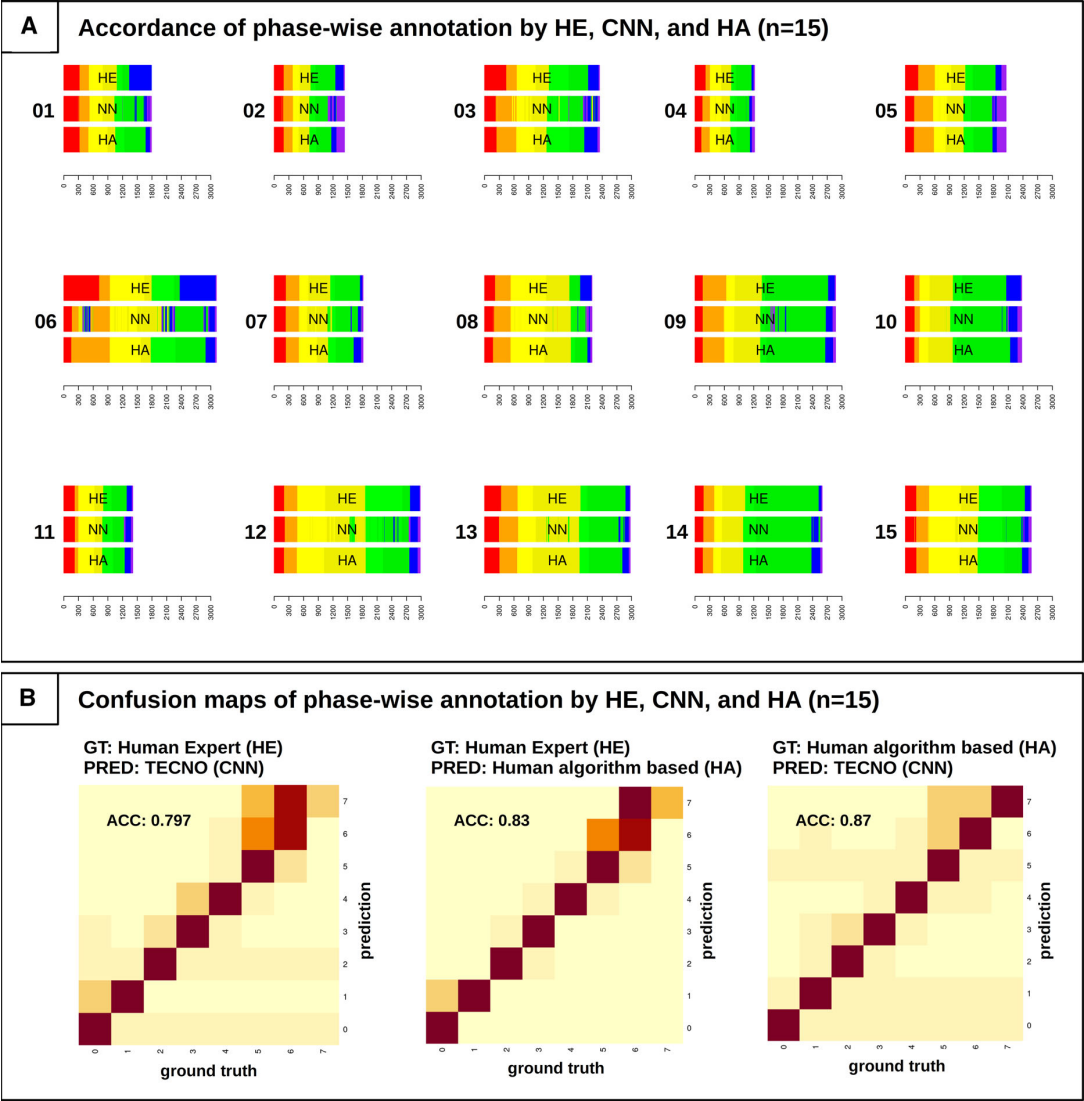
**a** annotation results of the 15 study surgeries by the Human Expert (HE), the Human Annotator algorithm based (HA), and the Neural Network TeCNO (CNN); **b** Confusion maps of the accordance of the three annotations. ACC: accuracy, GT: ground truth, PRED: prediction

The median phase length and IQR are shown in Fig. 4a. Overall, accuracy varied across the phases in relation to the HE as ground truth. In particular, CNN annotated with a low accuracy of 0.64 for phase 7, while HA performed much better with 0.99, but had a lower accuracy of 0.70 for phase 6. Overall, the human annotators generated smoother phases, as irregularities are prevalent in the CNN annotation, especially in the last half of the procedures and if their duration exceeded the IQR. Of course, a human annotator will not evaluate each single frame as a CNN does. Thus, he/she will specify a start and end point of a phase and assign all intervening frames to the particular phase. In this sense, the frame-by-frame annotation of the CNN might be more susceptible to interference. The overall accuracy of the CNN compared to HE was 0.797 (95%CI 0.793–0.802) and 0.83 (95%CI 0.826–0.834) for HA compared to HE. Thus, HA performed slightly more accordingly to the assumed ground truth HE, especially in the last two phases where it was difficult to distinguish between gallbladder removal and hemostasis. The time course of each procedure and confusion maps for the accordance are shown in Fig. 2.

### Phase flickering

**Fig. 3:**
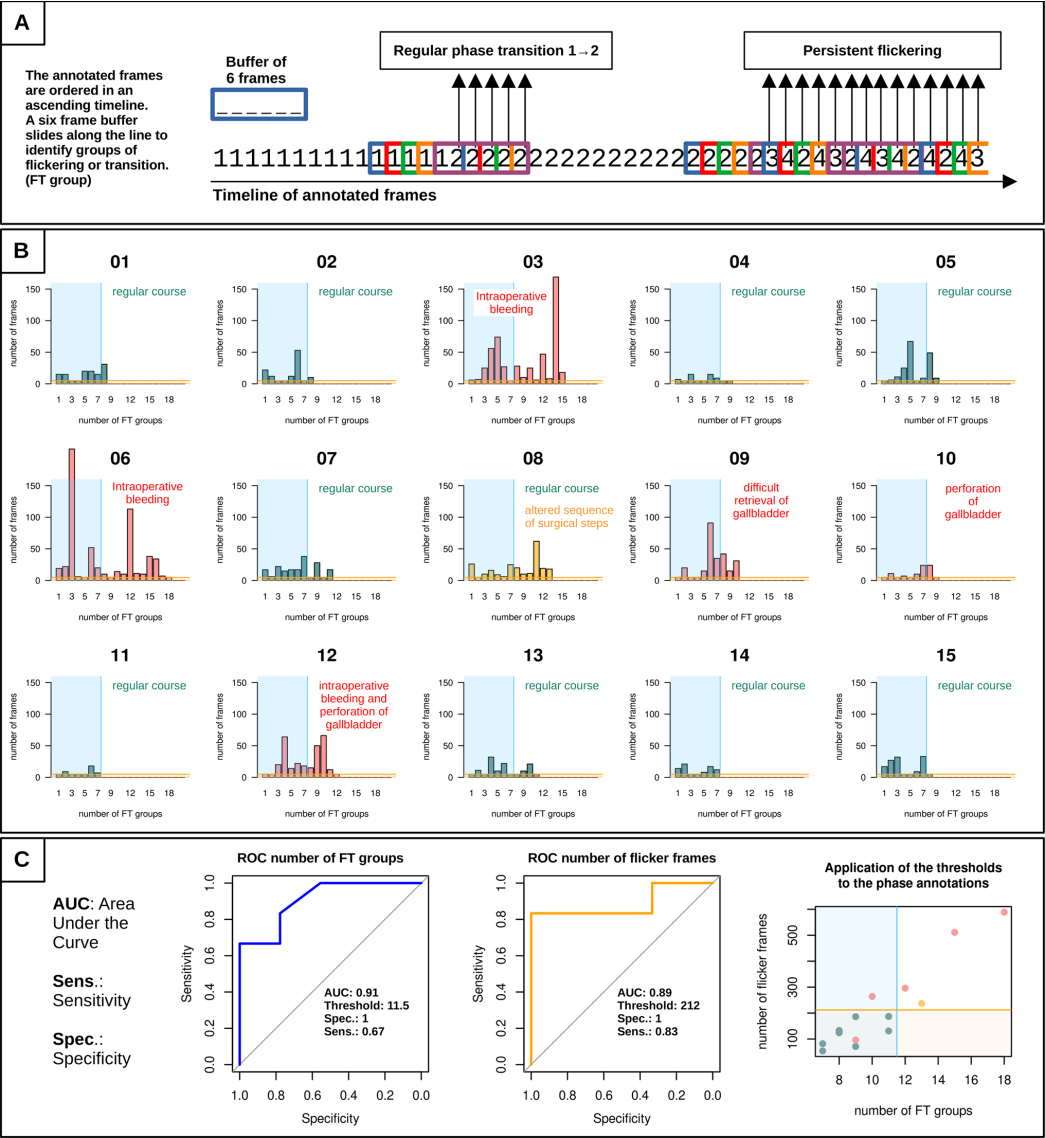
**a** In a self-written R script, a 6 frames wide buffer sliding along the ordered row of annotated frames is used to identify phase transitions or sequences of persistent flickering, both determined as FT groups (Flickering/Transition groups) **b** Analysis of the cholecystectomy records for the number of FT groups and comprehensive number of frames in these groups. The blue area in each plot defines an arbitrary cutoff of 7 FT groups, pretending a number of 8 phases and 7 transitions in case of a regular course. The orange area defines the estimated cutoff of 6 frames per FT group, again pretending a regular course with exclusively transitions and without flickering. **c** ROC analysis for the discrimination between regular and aberrant course by assessment of FT groups and number of concerned frames, the blue line in the scatter plot stands for the ascertained cutoff of 11.5 FT groups and the orange line for the cutoff of 212 flickering frames in all FT groups

Although the TeCNO CNN considers the time course of a surgical procedure, in several annotations, we recognized an outstanding interference with the aspect of some kind of flickering. (Fig. 2a *surgery 03, 06, 09, 12, 13*). Emerging the fact that this flickering appeared mainly in long surgeries, we analyzed these annotations in more depth and reassessed the concerned frames of each single surgery. We recognized that groups of persistent flickering often indicate complications such as bleeding or hampered retrieval of the gall bladder. (Fig. 3) To objectify the flickering, we wrote a script in the statistical framework R to identify flicker phases more easily. Technically, a buffer of 6 frames sliding along the row of annotated frames was used to decide whether a change in the annotated phase occurred or not. (Fig. 3a) Thereby, 3 relevant possible states of the loaded buffer can be defined: **1.** the buffer is filled with frame labels of the same kind (e.g. |1|1|1|1|1|1|1|) indicating no phase variation, **2.** the buffer is filled with two different labels of ascending order (e.g. |1|1|1|2|2|2|) indicating a regular transition of two phases, or **3.** the buffer is filled with more than two different labels with unsorted order (e.g. |2|3|1|1|2|4|) indicating a phase of flickering. Coherent phases of Flickering or Transition with an interval of maximum 10 s (‘FT groups’) were embraced and then statistically analyzed by their number and length per surgery. (Fig. 3b).

We discovered that a high amount of coherent incorrectly assigned frames and a high number of FT groups indicated complicated courses in 4 surgeries and a deviation from the order of surgical phases in 1 surgery. In 1 case a perforation of the gall bladder did not increase the amount and amplitude of FT groups as this adverse event had no significant impact on the course of the particular surgery. The ROC analysis of the number of FT groups and the number of aberrant frames revealed a critical value of 11.5 FT groups and of 212 incorrectly assigned frames per surgery as possible cutoff values. These two parameters achieved an area under the curve of 0.91 and 0.89 respectively. Their application to the 15 study videos identified 5 of the 6 surgeries with apparently divergent course due to complication.

### Final output

**Fig. 4:**
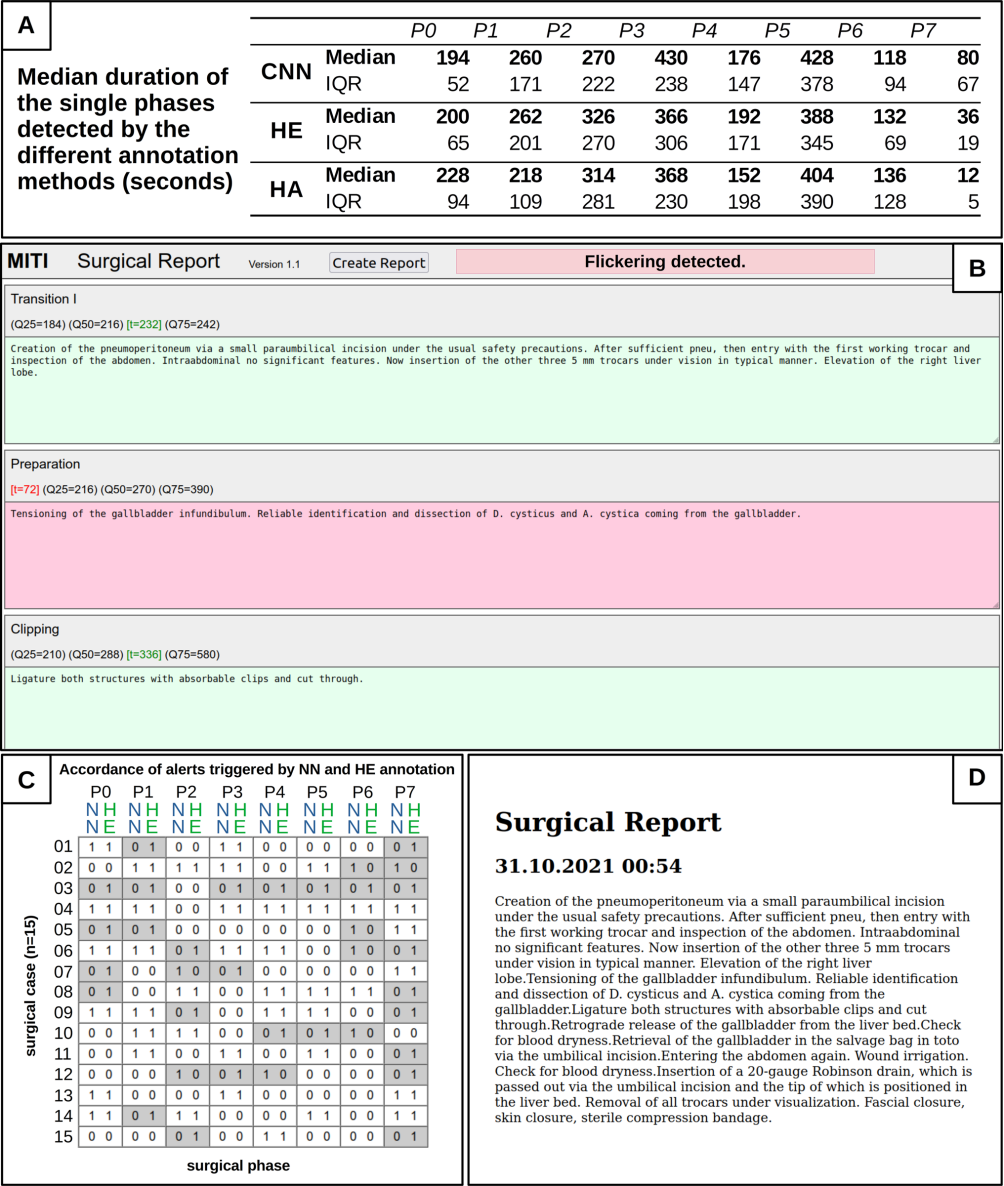
Creation of the final narrative yet structured surgical report with the MITI Surgical Report tool; **a** median values and interquartile ranges (IQR) of each phase detected by the Human Expert (HE), the algorithm based Human Annotator (HA), and the Convolutional Neural Network (CNN); **b** a deviation from the IQR of a specific phase causes an alert in the GUI (red background); **c** the accordance of alerting behavior based on HE annotation and CNN annotation was 70.8%; **d** the program returns a complete surgical report based on the annotations and comments by the surgeon

The final step of the herein presented workflow was the creation of a standardized surgical report based on the CNN’s output. The exclusively for this purpose programmed MITI Surgical Report tool was therefore provided with the phase durations (seconds) derived from its annotation. The accordance of alert behavior between CNN and HE was 70.8%. Furthermore, an alert was added in the header space of the GUI if a significant flickering was detected in the CNN’s output requiring comments on the suspected complication. The flickering alert was triggered if the critical value of 11.5 FT groups and/or 212 illogically assigned frames per surgery have been detected. (Fig. 4c) Finally, the completed surgical report was generated in a narrative design, based on the standard texts and additions by the surgeon. (Fig. 4d**).**

## Discussion

Based on the annotations achieved by TeCNO, we were able to provide a proof of concept for computer generated and manually completed surgical reporting in a preclinical scientific set up. The proposed workflow offers a standard surgical report to the surgeon and announces reliably, where possible adverse events or aberrance from standard course occurred. Thus, the surgeon only has to change the standard text if necessary. Otherwise, if a surgery is performed entirely in accordance with the standards, no further intervention by the surgeon is required. This principle of a documentation based on deviations from a standard course was already implemented in the approach of Just et al. by the use of automatically generated checklists which have to be completed by human interaction in real time. [19] In our case, during the surgery, no action by the surgeon or an observer is required. Moreover, we integrated an analysis of phase flickering and thus detected the occurrence of adverse events as bleeding and gallbladder perforation with a sensitivity of 67% for the number of FT groups and of 83% for the number of affected frames. The specificity for both parameters was estimated to be 1, suggesting that an adverse event is rather improbable if no flickering is detected. Regarding the annotation accordance of the CNN and the HE of 79.7%, there must be taken into account, that the original task was to assign one of the 8 phases to the video sequences. [11] As already mentioned, a human annotator will use another strategy than an CNN. He/she will first define the phase transitions and then will fill the frames between these timestamps with the annotation of the suitable phase, as assignment of each single frame to a certain phase would take many hours or even days for only one surgery. [20] Furthermore, the annotation of the CNN bases on an image recognition with respect to the temporal course. For this reason, in ambiguous constellations regarding time course and image content, the CNN may opt for a phase that does not make sense considering the original task but delivers additional information about intraoperative complications encoded in form of the above announced flickering. Therefore, the accordance of 79.7% cannot be seen as the only parameter to benchmark the performance of the CNN in that context. Although the assessment of ‘flickering’ at this time is only feasible to decide whether an adverse event or a rough aberrance from the standard procedure occurred or not, we estimate that the potential of a flickering analysis could go a lot further. With higher numbers of example videos in the inference group, statistical analysis of flickering patterns may be possible and could define certain types of complications or at least groups like ‘bleeding’, ‘perforation’, and ‘technical problems’ without having introduced them intentionally in the training process. In our proof of concept, we used the flickering solely to trigger an additional alert in the GUI’s header section.

A strength of our approach is the versatility of finally generated operative reports. While we exemplary proposed a narrative report in the last step of our workflow, even a more structured synoptic documentation is possible as the annotated phase lengths and flickering parameters can be the basis for each kind of final output, depending on users’ requirements regarding data extraction or detailed description of single steps of an operation. [21] Even hybrid reports uniting narrative and synoptic virtues are possible.

In our study, the laparoscopic cholecystectomy served as a highly standardized and approved procedure model for machine learning. [22] Of course a less structured surgery for example in an emergency setting may be much more challenging. But even if our approach becomes solely available for standard procedures as cholecystectomy or laparoscopic appendectomy in daily clinical use, it could reduce the documentation effort significantly. Certainly, our results confirm that it is mandatory to expand the basis for computer aided surgical documentation by further parameters, for example driven by vital sign sensors as well as other patient and procedure specific data, similar to the requirements of workflow recognition. [23] However, mere phase annotation seems not capable to reproduce the course of a surgery detailed enough to comply with the aim of retracing an entire procedure, as postulated in the introduction section. [1] A sage combination with other very promising approaches as real-time text recognition and keyword-augmented sequence prediction could even open up more complex and unpredictable procedures for automated reporting. [24]

While machine learning in terms of phase and instrument recognition proceeds at a breathtaking pace with accuracies of up to 92%, practical use cases in a daily clinical context are not yet fully uncovered. [9] The computer aided creation of surgical documentation is doubtless a promising application. Thus, next steps in development of an automated surgical reporting system are to analyze more surgeries in a prospective study design, and to identify more descriptors that contribute to a sustainable and rich in content documentation. Moreover, a common definition of what kind of information a surgical report should essentially contain is urgently needed, even to achieve international comparability and scientific assessment. Last but not least, the ideal design of final output is not thoroughly defined. Actually, a synoptic report seems to offer more advantages than a narrative text regarding information density and structure. [25] Nevertheless, we may not forget that a surgical documentation is primarily created to be read by human medical professionals with the intent to facilitate best patient care, and must not forfeit legibility as price for structure and machine interpretability.
